# Symptoms are an optimal target for orthostatic hypotension screening and management: an argument in favor

**DOI:** 10.1007/s10286-026-01213-4

**Published:** 2026-04-20

**Authors:** Jose Ricardo Lopez Castellanos

**Affiliations:** Department of Neurology, Medical University of South Carolina, Charleston, SC USA

**Keywords:** Orthostatic hypotension, Orthostatic intolerance

## Abstract

Orthostatic hypotension (OH), defined as a sustained reduction of ≥ 20 mmHg in systolic or ≥ 10 mmHg in diastolic blood pressure within 3 minutes of standing, represents one of the most clinically significant manifestations of autonomic failure. Beyond its hemodynamic definition, OH is associated with disabling symptoms, falls, syncope, reduced quality of life, increased healthcare utilization, and excess mortality. In clinical practice, management often requires balancing objective blood pressure measurements with the patient’s lived experience of orthostatic intolerance. This viewpoint argues that symptoms represent an appropriate and clinically meaningful target for screening and management of OH. Three central assumptions support this perspective. First, patients are reliable reporters of orthostatic symptoms and clinicians are capable interpreters of these reports. Validated patient-reported outcome measures, such as the Orthostatic Hypotension Questionnaire (OHQ), demonstrate that symptom burden and functional impairment can be reproducibly quantified and that clinically meaningful changes can be detected. Second, although the relationship between orthostatic blood pressure changes and symptoms is not absolute, evidence supports a clinically relevant association, with symptomatic individuals often experiencing greater cerebral hypoperfusion when upright. Third, symptoms serve as a practical proxy for meaningful OH-related outcomes, including functional independence, fall risk, and quality of life, and have been accepted as primary endpoints in pivotal clinical trials. A symptom-centered framework complements objective hemodynamic assessment by contextualizing physiological findings within patients’ functional experience. Integrating symptom reporting with orthostatic measurements provides a pragmatic, patient-centered approach to screening, treatment decisions, and evaluation of therapeutic response in OH.

## Introduction

Orthostatic hypotension (OH), as defined by consensus criteria, is a drop of at least 20 mmHg in systolic or 10 mmHg diastolic blood pressure within 3 min of assuming an upright position and represents one of the most clinically consequential manifestations of autonomic failure [1]. It is associated with falls, syncope, impaired quality of life, increased hospitalization, and excess mortality [2, 3]. For patients, OH is not just a numerical change in blood pressure, as it may present in the form of dizziness, visual dimming, fatigue, presyncope or syncope, cognitive clouding, or the more subtle but persistent loss of independence and confidence when ambulating [4].

In clinic, managing OH is often a balancing act. We aim to identify those patients at risk, reduce disabling symptoms, and preserve their function, while being mindful of potential treatment-related complications, such as supine hypertension. Formally, OH has been defined by hemodynamic criteria, but clinical decisions are driven by symptoms. Our patients seek medical care because they feel unwell when upright. Often, we feel compelled to escalate therapy specifically because symptoms interfere with their daily life and treatments are deemed successful not when blood pressure normalizes but when patients are able to stand up, walk, and perform their daily activities safely and with greater confidence [1, 3].

Therefore, a central question for the management of OH is not whether objective blood pressure measurements are necessary, which they clearly are, but whether their symptoms represent an optimal target for screening and management. In this viewpoint article, I argue in favor of three core assumptions: first, that patients are reliable reporters of OH symptoms and physicians are reliable interpreters of those reports; second, that a clinically meaningful relationship exists between orthostatic hemodynamics and symptoms; and third, that symptoms serve as an appropriate and useful proxy for OH-related outcomes. Far from arguments emphasizing asymptomatic OH and the limitations of symptoms versus blood pressure correlation, I contend that symptom-based assessment best reflects clinical relevance, patient-centered goals, and should guide evidence-based management of OH.

## Assumption 1: patients are reliable reporters and physicians are reliable recorders

In the clinical setting, OH is recognized while obtaining a story and not just by reading from a sphygmomanometer. We can sense the lack of confidence and independence as patients describe their symptoms; namely, feeling lightheaded while standing, needing to take a break before walking, avoiding showers or meals due to dizziness, or experiencing near-syncope. Thus, orthostatic symptoms are the clinical signal of an inadequate baroreflex response to upright posture [3].

Our primary goal when managing OH is not normalization of standing blood pressure but the relief of symptoms that interfere with quality of life, a principle supported by consensus statements and reflected in current clinical trial design [1, 5, 6]. This approach is based on evidence showing that the magnitude of blood pressure drop does not consistently correlate with functional impairment or severity of symptoms [3, 4]. For example, two patients with identical orthostatic blood pressure drops may experience immensely distinct levels of disability. Their symptoms, rather than numbers alone, define clinical relevance.

The availability, use, and performance of validated patient-reported outcome (PRO) measures are usually overlooked by existing concerns that patients are unreliable symptom reporters. The Orthostatic Hypotension Questionnaire (OHQ), which includes the Orthostatic Hypotension Symptom Assessment (OHSA) and the Orthostatic Hypotension Daily Activity Scale (OHDAS), was developed to translate patient subjective experiences into reproducible data. This questionnaire underwent rigorous validation against functional and quality-of-life measures, and when used as a primary endpoint in randomized controlled clinical trials, the OHQ demonstrates that patients can reliably quantify their symptom burden in a consistent and clinically meaningful way [4, 7].

Confidence in symptom reporting has been further strengthened after the recent establishment of a minimal clinically important difference (MCID) for the OHQ [8]. For example, a 2- to 3-point improvement in the single-item OHSA1 corresponds to a meaningful patient-perceived benefit, while a 1-point worsening translates into clinical deterioration. The establishment of a MCID links symptom changes that patients recognize as important into palpable outcomes, transforming symptom reporting from a descriptive exercise into an actionable clinical metric.

Beyond the OHQ, we widely utilize blood pressure diaries along with other autonomic symptom scales (such as COMPASS-31, SCOPA-AUT, and MSA-QoL, etc.) in both research and clinical practice. Studies have demonstrated a reasonable clinical concordance between patient and caregiver reports, supporting the reliability of symptom assessment even in neurodegenerative conditions [5, 9]. Most importantly, symptom reporting, in the form of diurnal variations, exertional intolerance, postprandial hypotension, medication side effects, near-syncope, or activity avoidance, adds context and timing that is otherwise invisible during a single blood pressure measurement [3, 4, 9].

The emphasis on the prevalence of asymptomatic OH and hypotension unawareness frequently arise in opposing arguments, but neither of these invalidates symptom-based care. These statements highlight the heterogeneity of OH and reinforce the importance of symptoms as markers of functional impact when obtaining real-world data.

In clinical practice, we rely on the integration of symptom reporting, orthostatic vitals, ambulatory blood pressure monitoring, and autonomic testing results. Therefore, the intention is not for symptoms to replace objective measures but to give them meaning [10].

## Assumption 2: there is a clinically meaningful relationship between orthostatic hemodynamics and OH symptoms

In contrast to other cardiovascular conditions, we treat OH because patients perceive its debilitating consequences when upright, and not simply because of a pathological blood pressure fall. At least three studies have shown that, on average, the blood pressure in symptomatic patients crosses beyond critical perfusion thresholds more frequently than in those who remain asymptomatic, particularly in neurogenic OH [11–13]. Though this relationship is not absolute, it is still clinically meaningful. OH symptoms tend to appear after exceeding cerebral autoregulatory reserve mechanisms, which can help us identify patients at the highest risk for falls, syncopal events, and functional impairment [14].

Cerebral hemodynamic data provides further support to the biology of this relationship. A recent systematic review and meta-analysis of transcranial Doppler studies by Baker et al. demonstrated a linear association between reductions in upright mean arterial pressure and decreases in cerebral blood flow in neurogenic OH across included studies [15]. Patients with symptomatic OH experienced a greater magnitude of cerebral hypoperfusion, providing a mechanistic link between clinical experience and orthostatic blood pressure physiology.

We cannot forget about an essential factor: patient-to-patient variability. Some patients tolerate a substantial blood pressure drop or cerebral blood flow reduction without symptoms [16–18]. Nonetheless, clinical medicine rarely relies solely on a perfect correlation. Symptoms function as integrative signals that reflect both cerebral autoregulation and hemodynamics, as well as interactions with medications, other comorbidities, and daily activity demands. This integrative quality makes symptoms essential in management strategies in the real world.

We need feedback from symptoms experienced by patients to modify treatment of both pharmacological and non-pharmacological strategies. We usually adjust medications such as midodrine and droxidopa, in conjunction with fluid and salt loading, compression garments, and bed tilt-up, based on a delicate balance between benefits and the appearance of adverse effects [3]. The goal should be to improve daily functioning when upright, and not blood pressure normalization alone. In this sense, PROs provide the most clinically meaningful outline for balancing both efficacy and safety, particularly in patients at risk of developing supine hypertension.

## Assumption 3: symptoms are a useful proxy for OH-related outcomes

The value of any clinical target depends on whether the outcomes matter. In OH, we can precisely capture these outcomes: from the ability to stand and walk independently to the performance of daily activities when upright. In patients with OH, the symptom burden determines the preservation or loss of independence [4, 9].

The US Food and Drug Administration (FDA) accepts patient-reported symptom improvements as a valid, and sometimes, even preferred, primary endpoint for therapies targeting OH [19]. As a perfect example, the OHQ served as the principal outcome measure in the pivotal trials that led to the approval of midodrine and droxidopa [3–6]. To this author’s knowledge, no other objective cardiovascular autonomic biomarker has undergone an equivalent regulatory scrutiny or a similar responsiveness to treatment.

Two observational studies have shown that adverse outcomes, such as falls or cognitive impairment, may occur even if individuals are asymptomatic [20, 21]. On the basis of this important observation, one should strive for the complementary use of objective measures, instead of just relinquishing symptom-based management. This is an opportunity to identify when OH is clinically consequential and warrants a more aggressive intervention and not just an argument about whether symptoms predict adverse outcomes [22, 23].

When comparing individuals with asymptomatic versus symptomatic OH, the latter has been associated with greater healthcare utilization, increased risk of syncopal events, reduced quality of life, and higher overall mortality [1, 3, 9]. Symptom burden functions as a realistic proxy for disease impact, guiding initiation, escalation, or de-escalation of therapy [18]. In this sense, symptoms are practical clinical targets. We can assess symptoms quickly, repeatedly, and longitudinally across diverse care modalities. Technology-objective measures are valuable, but they are not yet universally accessible. A symptom-driven assessment ensures that OH management remains patient-centered and adjustable based on feedback.

## Clinical implications

In practice, our screening begins by looking for orthostatic intolerance symptoms, followed by physiological confirmation of positive orthostatic blood pressure measurements that are consistent with expert recommendations. This symptom-centered approach provides a clear and pragmatic framework for OH management [3, 4]. Severity, frequency, and functional impact of symptoms, rather than blood pressure thresholds alone, should guide treatment decisions. It is essential to educate both patient and family members to accurately capture and report orthostatic intolerance symptoms, helping clinicians with the adequate interpretation of these results. Adjustment of therapies aims to improve upright function while minimizing side effects, particularly supine hypertension. The use of the OHQ and other patient-related outcome measures helps clinicians assess treatment response, adjust interventions, and empower patients towards their plan of care.

The integration of symptoms and clinical management should align diagnostic reasoning, therapeutic goals, and outcome assessments to what matters the most to patients: the improvement in daily function, safety, and quality of life.

## Conclusion

We define orthostatic hypotension by clinical blood pressure criteria, but patients experience OH through symptoms. Objective measurements remain essential for OH diagnosis and risk stratification, but symptoms are the reason patients seek care, determine how clinicians make treatment decisions, and evaluate therapy success. This article supports the view that symptoms are not just acceptable targets for OH screening and management but the most clinically relevant outcome target. A symptom-based approach offers a feasible, validated, and clinically meaningful framework for the management of OH. The presence of asymptomatic OH and its somber counterpart, supine hypertension, calls for a complementary approach in which objective measures gauge risk and symptoms guide clinical management, preserving what matters the most to patients: independence.
